# Development of GUI-Driven AI Deep Learning Platform for Predicting Warpage Behavior of Fan-Out Wafer-Level Packaging

**DOI:** 10.3390/mi16030342

**Published:** 2025-03-17

**Authors:** Ching-Feng Yu, Jr-Wei Peng, Chih-Cheng Hsiao, Chin-Hung Wang, Wei-Chung Lo

**Affiliations:** 1 Department of Mechanical Engineering, National United University, Miaoli 360302, Taiwan; 2 Electronic and Optoelectronic System Research Laboratories, Industrial Technology Research Institute (ITRI), Hsinchu 30010, Taiwan; jrweipeng@itri.org.tw (J.-W.P.); zchsiao@itri.org.tw (C.-C.H.); jerry_wang@itri.org.tw (C.-H.W.); lo@itri.org.tw (W.-C.L.)

**Keywords:** AI prediction platform, fan-out wafer-level packaging (FOWLP), warpage prediction, graphical user interface (GUI), deep learning, finite element analysis (FEA)

## Abstract

This study presents an artificial intelligence (AI) prediction platform driven by deep learning technologies, designed specifically to address the challenges associated with predicting warpage behavior in fan-out wafer-level packaging (FOWLP). Traditional electronic engineers often face difficulties in implementing AI-driven models due to the specialized programming and algorithmic expertise required. To overcome this, the platform incorporates a graphical user interface (GUI) that simplifies the design, training, and operation of deep learning models. It enables users to configure and run AI predictions without needing extensive coding knowledge, thereby enhancing accessibility for non-expert users. The platform efficiently processes large datasets, automating feature extraction, data cleansing, and model training, ensuring accurate and reliable predictions. The effectiveness of the AI platform is demonstrated through case studies involving FOWLP architectures, highlighting its ability to provide quick and precise warpage predictions. Additionally, the platform is available in both uniform resource locator (URL)-based and standalone versions, offering flexibility in usage. This innovation significantly improves design efficiency, enabling engineers to optimize electronic packaging designs, reduce errors, and enhance the overall system performance. The study concludes by showcasing the structure and functionality of the GUI platform, positioning it as a valuable tool for fostering further advancements in electronic packaging.

## 1. Introduction

Semiconductor manufacturing has traditionally been driven by the pursuit of smaller, faster, and more energy-efficient devices. However, as transistor dimensions approach near-atomic scales, fundamental challenges—such as quantum effects, heat dissipation, and issues with the power efficiency—are emerging, thereby threatening the continued progress predicted by Moore’s Law [1]. In light of these physical limitations, the industry has increasingly embraced the “More than Moore” paradigm, which shifts the focus from mere miniaturization to heterogeneous integration through advanced packaging technologies [2,3,4,5,6,7,8]. By integrating multiple functionalities—processing, memory, sensing, and communication—into a single compact package, this approach not only enhances system performance but also mitigates the challenges associated with further transistor scaling. A prominent technology within this paradigm is fan-out wafer-level packaging (FOWLP), which offers several key advantages, including a higher input/output (I/O) density, a reduced overall form factor, and improved electrical performance [2,3,4,5,6,7,8]. These features make FOWLP particularly well suited for heterogeneous system integration, where the integration of diverse components into a unified package reduces the system complexity, lowers manufacturing costs, and improves efficiency. Nevertheless, despite its growing adoption and widespread recognition, FOWLP faces significant technical challenges. Key issues include yield optimization, reliability enhancement, thermal management, and—most critically—the control of warpage during manufacturing. Warpage may occur at various stages of the FOWLP process, potentially leading to component misalignment, inaccuracies in material handling, and registration errors during assembly. These deformations can result in reduced yields, increased production costs, and an overall diminished efficiency [6,9].

To address these challenges, a comprehensive understanding of warpage behavior at the initial design stage is imperative. The literature documents numerous studies that utilized finite element analysis (FEA) to characterize and manage warpage during FOWLP fabrication [6,7,8,9,10]. Compared to experimental methods, theoretical analyses via FEA offer greater efficiency and cost-effectiveness while providing deeper insights into the underlying physical mechanisms. For example, Cheng et al. [9] investigated process-induced warpage in molded wafers for chip-first, face-down FOWLP, developing simulation methodologies that evaluate shrinkage, non-linearity, and gravitational effects, with inline measurements corroborating their findings on the geometric factors influencing warpage. Similarly, Yu et al. [6] implemented a process-dependent simulation that integrated non-linear FEA with an element birth and death technique to assess warpage in FOWLP technologies featuring through-silicon via array interposers. Their work, combining experimental validation with a response surface methodology, demonstrated the practical feasibility of warpage suppression strategies. In addition, Lee et al. [10] introduced a multilevel simulation strategy that employs an equivalent material method for modeling fine redistribution layers and micro bumps, achieving prediction errors below 5%.

To address these challenges and reduce prediction uncertainty and modeling errors made by less experienced engineers, researchers have increasingly integrated simulation with machine learning, leveraging recent advancements in computer technologies and machine learning algorithms to expand the methodological toolbox available to the semiconductor industry. Various techniques, including support vector regression (SVR) [11], the random forest (RF) method [12], gradient boosting regression (GBR) [13], the K-nearest neighbors (KNN) [14], and kernel ridge regression (KRR) [15], have been applied to model performance parameters in microelectronic packaging. These techniques can process extensive amounts of structured data to reveal intricate interdependencies and generate predictive insights that optimize design processes. For instance, GBR effectively manages non-linear relationships, while SVR leverages high-dimensional feature spaces to capture subtle patterns. In contrast, the KNN is valuable for delineating ambiguous decision boundaries, and both KRR and the RF method demonstrate robust performance in handling noisy data and complex variable interactions.

Complementing these machine learning methods, deep learning architectures have further enhanced predictive capabilities in microelectronic packaging. Models such as recurrent neural networks (RNNs) [16], gated recurrent units (GRUs) [17], multilayer perceptrons (MLPs) [18], and long short-term memory networks (LSTM) [19,20] have proven effective in capturing temporal dependencies and forecasting long-term performance trends. These models are particularly well suited for sequential and time-series data analysis, as demonstrated in practical applications. For example, Law et al. [21] developed an artificial neural network (ANN) model to predict the thermal behavior of quad flat no-lead (QFN) packages, while Subbarayan et al. [22] proposed a reliability prediction model for ball grid array (BGA) packages. More recently, Hsiao and Chiang [12] employed the RF method to forecast the reliability lifespan of wafer-level packages (WLPs) through FEA simulations, and Panigrahy et al. [23] compared multiple AI-assisted design-on-simulation algorithms to optimize WLP reliability. Additionally, Kuo et al. [24] and Cheng et al. [25] applied SVR- and ANN-based models, respectively, to predict WLP reliability and flip-chip chip-scale package warpage.

In this study, a suite of machine learning techniques—including SVR [11], the RF method [12], GBR [13], the KNN [14], and KRR [15]—is rigorously compared with deep learning models such as RNNs [16], GRUs [17], MLPs [18], and LSTM [19,20]. The algorithm demonstrating the closest alignment with FEA data was selected for integration into a warpage prediction platform, ensuring optimal accuracy and reliability for practical applications. An FEA-driven process modeling approach was also implemented, which accounted for the viscoelastic properties of the epoxy molding compound (EMC) and the temperature-dependent thermal–mechanical characteristics of FOWLP materials. This integrated framework enhances the understanding of material influences on warpage behavior during manufacturing, with the simulated results validated against experimental data to confirm both theoretical robustness and practical relevance. Furthermore, a detailed parametric analysis was conducted to identify the key factors influencing warpage, which were then incorporated into the predictive model to accommodate varying material and process conditions. The final model was integrated with a user-friendly graphical user interface (GUI) and evaluated using an independent validation dataset. The GUI enables real-time parameter adjustments and instant predictions, thereby streamlining the design process for engineers and enhancing the overall efficiency and reliability of FOWLP systems. Collectively, these advancements represent a significant step forward in microelectronic packaging design. By combining advanced FEA-driven simulations with sophisticated machine learning and deep learning techniques, and by integrating these tools into an intuitive GUI, this study provides both a theoretical framework and a practical tool for overcoming the challenges of warpage in FOWLP. This integrated approach not only refines the accuracy of warpage predictions but also supports the ongoing evolution of semiconductor technologies in the face of ever-increasing demands for performance and energy efficiency.

## 2. Structure and Fabrication Process of FOWLP

**Figure 1:**
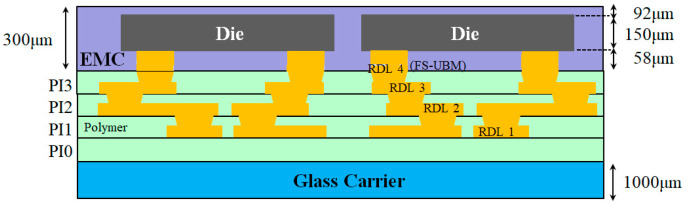
The FOWLP assembly.

The structure of the FOWLP under investigation is illustrated in Figure 1. The fabrication process of FOWLP consists of several sequential steps that transform a glass carrier and silicon die into a complete package. The process begins with the deposition of a polyimide (PI) layer onto a glass carrier. This PI layer serves as the foundation for the subsequent buildup of electrical and structural layers. After the PI layer is deposited, the assembly is subjected to a curing process at a temperature of approximately 210 °C. This curing step firmly bonds the PI layer to the glass carrier and prepares the surface for the following process steps. After the PI layer is cured, the next stage involves the formation of redistribution layers (RDLs). In this stage, metal, typically copper, is deposited onto the cured PI surface. The metal is then patterned using photolithography to form fine wiring that will later interconnect the silicon die with external circuitry. At the same time, dielectric materials are deposited to provide electrical insulation between the metal lines. This stage is critical because it creates a routing network that enables the transmission of signals in the final package.

Once the RDLs are established, the process continues with the deposition of an under-bump metallization (UBM) layer on the silicon die. The UBM is a thin metallic coating applied to the areas of the die where connections are to be made. This deposition is carried out using physical vapor deposition techniques, and the metal is then patterned to align with the intended connection points. The UBM layer serves as the interface for the copper pillar bumps used later in the die bonding process. The subsequent stage is the die bonding process. In this step, the silicon die is carefully aligned and mounted onto the substrate that already contains the patterned RDLs. The bonding is accomplished by placing copper pillar bumps on the designated bond pads that connect the die to the substrate. The entire assembly is heated to around 260 °C to reflow the solder contained within the copper pillar bumps. This controlled heating process allows the solder to melt and form strong joints that secure both the electrical and mechanical connections between the die and the substrate.

After the die bonding stage is complete, the next fabrication step is the application of a liquid EMC. The EMC is dispensed over the bonded die and surrounding areas to form a protective layer that encapsulates the components. Following its application, the EMC undergoes a curing process at a temperature of approximately 150 °C. This curing step solidifies the compound and reinforces the overall assembly by providing mechanical support to the package. Once the encapsulation is complete, the package enters the singulation stage. During singulation, the completed wafer is cut or laser-diced into individual FOWLP units. This step separates each package from the wafer and ensures that the structural integrity of each unit is maintained for further testing and integration into electronic systems. Throughout the entire fabrication process, strict environmental controls and precise process parameters are maintained. From the initial deposition of the PI layer to the formation of RDLs, UBM deposition, die bonding, encapsulation, and finally singulation, each step requires careful monitoring to guarantee reproducibility and reliability. The process is typically carried out in a clean room setting to minimize contamination and defects, which is essential for producing high-quality FOWLP units.

## 3. Theoretical Frameworks of Machine Learning and Deep Learning

In this study, a combination of both machine learning and deep learning techniques was utilized to perform a comprehensive analysis and prediction of complex datasets, addressing the challenges inherent in capturing nuanced patterns within diverse data types. The machine learning approaches employed in this investigation included SVR [11], the RF method [12], GBR [13], the KNN [14], and KRR [15]. Each of these methods brings distinct capabilities and strengths to the table, particularly in their ability to process structured data, which are a common characteristic of many real-world datasets. These algorithms excel in creating models that are capable of uncovering intricate relationships within data, and they vary in their approach to learning from the input variables. For instance, GBR is highly effective in handling non-linear relationships by sequentially building a series of models that correct the errors of previous iterations, whereas SVR focuses on constructing hyperplanes in a high-dimensional space to best separate the data points, offering robust performance even in complex scenarios. The KNN, on the other hand, is valuable for its simplicity in classification and regression tasks, utilizing distance metrics to make predictions based on the nearest neighbors in the feature space, while KRR merges the strengths of ridge regression with kernel methods to handle non-linearity. In addition, the RF method is recognized for its ensemble learning approach, which combines multiple decision trees to improve prediction accuracy, reduce overfitting, and handle a diverse range of input data types. This method is particularly advantageous when working with large datasets that contain noisy or unbalanced data, as the averaging of multiple trees mitigates the risk of overfitting, providing stable and reliable predictions.

Furthermore, deep learning techniques were also incorporated into the analysis, including RNNs [16], GRUs [17], MLPs [18], and LSTM [19,20]. These models are inherently more complex than traditional machine learning algorithms, as they are designed to automatically learn and extract features from unstructured data, such as time-series or sequential data. Their architecture allows them to recognize patterns across time steps, making them particularly well suited for tasks that require temporal dynamics, such as predicting trends in financial markets, understanding user behavior over time, or forecasting demand in supply chain management. The recurrent nature of RNNs allows them to maintain a memory of previous inputs, enabling the model to make more informed predictions based on past information. However, due to issues such as vanishing gradients, GRUs and LSTM were developed as advanced variants to overcome these limitations by introducing gating mechanisms that better control the flow of information through the network. These gated units are crucial for enabling the model to focus on relevant time steps, filtering out irrelevant information and thereby improving prediction accuracy.

In particular, LSTM networks are highly effective in managing long-range dependencies within sequential data, offering a distinct advantage over traditional RNNs in applications that require the retention of information across extended sequences. This makes LSTM networks particularly suitable for complex tasks such as speech recognition, language translation, and time-series forecasting, where the relationships between data points may span long intervals. GRUs offer a more streamlined architecture than LSTM networks, reducing computational complexity while still retaining the ability to capture temporal dependencies. This makes GRUs an attractive option for scenarios where computational efficiency is critical yet the task still demands the ability to model sequential dependencies with high accuracy.

MLPs, though not explicitly designed for sequential data, provide a versatile framework for handling both regression and classification tasks. As a feedforward neural network, an MLP operates by passing information through multiple layers of interconnected nodes, allowing the network to learn hierarchical representations of the data. This approach is particularly powerful when working with high-dimensional datasets, as it enables the model to discover complex relationships between input features and target variables. While MLPs are often used for structured data, they can also be applied to unstructured data, particularly when combined with other models or used as part of a larger ensemble. These deep learning models, with their capacity to process vast amounts of data and their ability to automatically learn complex feature representations, significantly enhanced the accuracy and robustness of the predictions generated in this study. By capturing intricate patterns that might be overlooked by traditional machine learning algorithms, these models contributed to a more nuanced understanding of the data, ultimately leading to more reliable and informed decision-making processes. The combination of both machine learning and deep learning methodologies thus provided a comprehensive toolkit for tackling the challenges presented by complex datasets, ensuring that the models developed in this study are both flexible and capable of delivering high-performance predictions across a variety of applications.

## 4. Finite Element Analysis (FEA) Model for FOWLP

**Figure 2:**
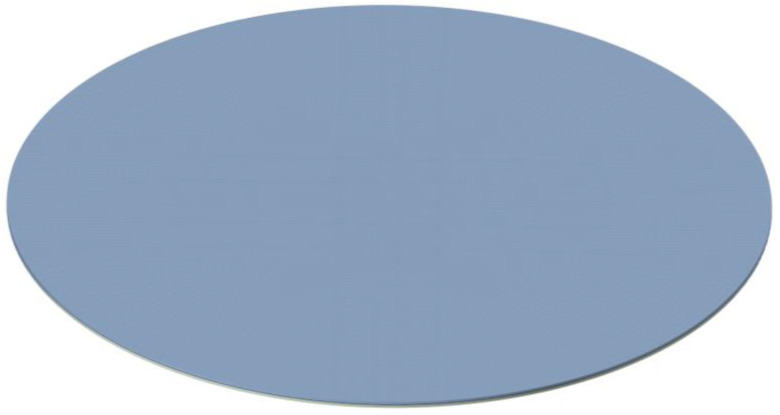
Three-dimensional FEA model of the FOWLP.

**Figure 3:**
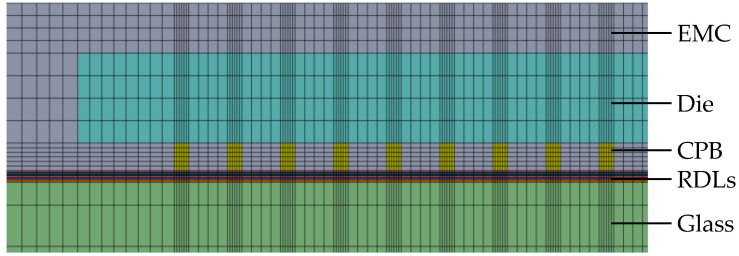
The detailed FEA model of the FOWLP.

**Figure 4:**
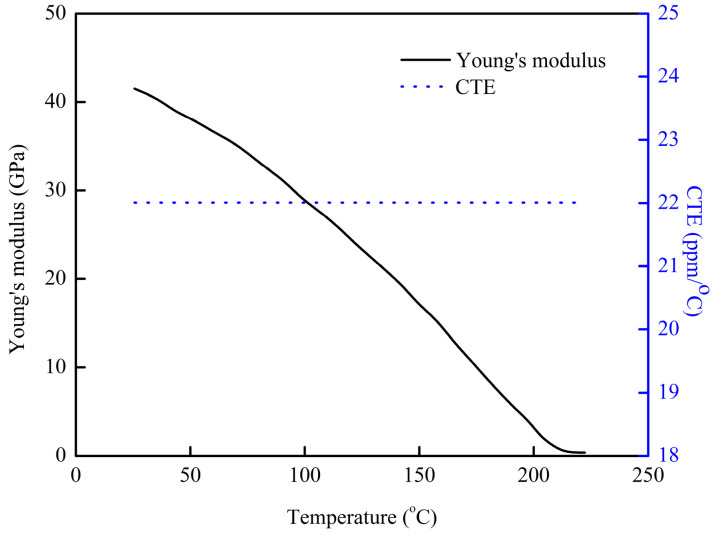
Thermal–mechanical properties of SAC 305 [25].

A comprehensive FEA model was developed, incorporating the ANSYS element birth and death technique alongside non-linear FEA, to provide an accurate assessment of the warpage behavior observed in FOWLP during its fabrication process. As illustrated in Figure 2, the model represents a fully three-dimensional simulation of the FOWLP structure. In order to eliminate the possibility of rigid body motion, the displacement of the nodes located at the center of the bottom surface of the glass carrier is constrained. The detailed composition of the FEA model, depicted in Figure 3, includes critical components such as RDLs, the EMC, CPBs, silicon dies, and the glass carrier. The mesh was constructed using hexahedral solid elements (ANSYS SOLID185), resulting in approximately 457,272 elements and 498,726 nodes in total for the entire FOWLP structure, ensuring a high level of precision in the warpage simulation. Table 1 shows the material properties of the WLP model. It is crucial to highlight that all materials, with the exception of the solder balls, exhibit linear behavior and are not influenced by temperature variations. The material properties of the SAC 305 solder balls, on the other hand, demonstrate non-linear behavior and are highly dependent on the temperature, as shown in Figure 4 [25]. While the EMC is modeled as a linearly viscoelastic material, all other materials are treated as linearly elastic, isotropic, and temperature-dependent, thereby ensuring an accurate representation of their mechanical responses in the simulation.

EMC materials are critical in determining the thermal–mechanical performance of electronic packaging systems [26]. These materials typically exhibit viscoelastic characteristics that are influenced by the temperature, time, and strain rate, leading to complex behaviors such as creep, stress relaxation, and hysteresis. The viscoelastic relaxation behavior of EMC materials is commonly modeled using a generalized Maxwell framework, which consists of multiple Maxwell elements and an independent spring arranged in parallel. This approach effectively captures the relaxation dynamics and is frequently represented through a Prony series expansion in the frequency domain, which provides an accurate fit to experimental relaxation data.

(1) $$E(\omega )=E_{0}(g_{\infty }+\sum_{i=1}^{N}g_{i}\frac{\tau_{i}^{2}\omega^{2}}{1+\tau_{i}^{2}\omega^{2}})$$

*E*(*ω*) denotes the relaxation modulus of the entire model, *g_i_* the weight factor of the *i*th Maxwell element, $g_{\infty }$ the long-term fully relaxed weight factor, *ω* the frequency, *τ_i_* the relaxation time, and *N* the total number of Maxwell elements.

The time and temperature dependence of the mechanical properties of a viscoelastic material can be correlated using the time–temperature superposition principle (TTSP). More specifically, the TTSP suggests that the relaxation curve of a viscoelastic material at a specific temperature can be employed as a reference for further characterizing the relaxation curves at other temperatures by conducting a horizontal translation of the reference relaxation curve in the logarithmic time domain. The temperature translation factor *λ_T_* is normally approximated using an empirical relationship, the so-called Williams–Landel–Ferry (WLF) equation:

(2) $$\log_{10}\lambda_{T}=\frac{-\kappa_{1}+(T-T_{r})}{\kappa_{2}+(T-T_{r})}$$

**Figure 5:**
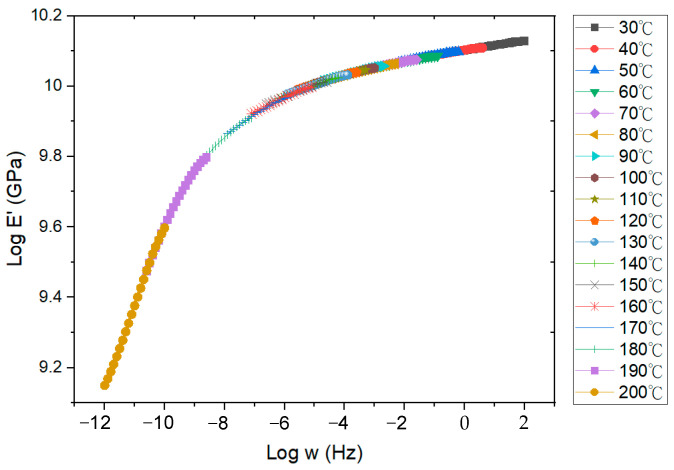
Established reference master curve of relaxation modulus and its Prony series curve.

**Figure 6:**
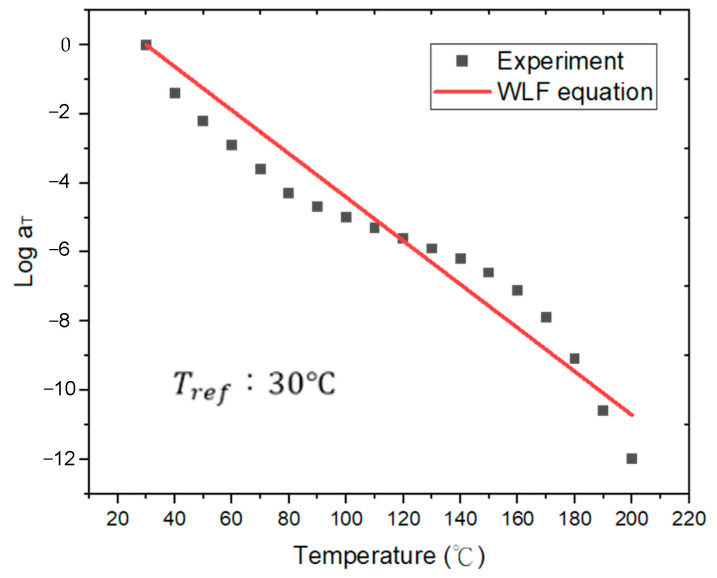
Shift factor (*a_T_*) and fitted result with the WLF equation.

*κ*_1_ and *κ*_2_ are identified as curve-fitting coefficients, with *T_r_* representing the reference temperature. The master curve of the relaxation modulus at this reference temperature can be derived by shifting the experimentally obtained frequency-dependent storage moduli measured at various temperatures along the time axis, utilizing the temperature-dependent translation factors *λ_T_*. Through the use of relaxation modulus data obtained under different isothermal conditions under 1% applied strain [9], a reference master curve was constructed at the glass transition temperature of the EMC, as depicted in Figure 5. The coefficients *g_i_* and *τ_i_* for the 20-term Prony series model, which were used to fit this master curve, are presented in Table 2. Moreover, using the shift factor as a temperature function, as illustrated in Figure 6, the fitted WLF model coefficients, *κ*_1_ = 6.311 × 10^7^ and *κ*_2_ = 1.001 × 10^9^, were determined for the translation factors, providing a temperature-dependent characterization.

## 5. Results and Discussion

### 5.1. Characterization of Process-Induced Warpage of FOWLP

In this study, an experimental approach using the Shadow Moiré measurement technique was meticulously employed to validate and confirm the accuracy of the FEA simulations for FOWLP. This technique, known for its high precision in measuring warpage and deformation, played a pivotal role in ensuring that the simulation results aligned closely with real-world experimental outcomes. By leveraging the capabilities of the Shadow Moiré method, this study aimed to provide a robust and reliable validation framework, ensuring the predictive models are not only theoretical but also applicable to practical scenarios in semiconductor packaging.

**Figure 7:**
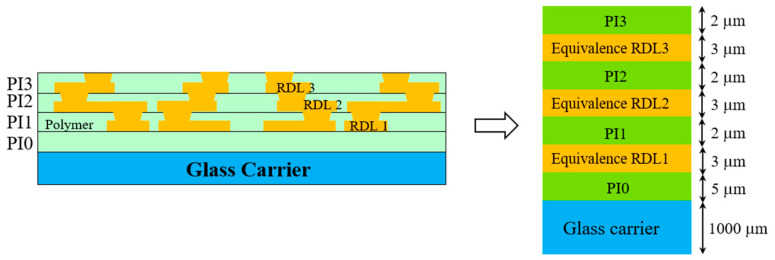
Schematic of the equivalent RDL.

Figure 7, presented in this study, offers a clear schematic representation of the RDLs within the FOWLP structure, key components that play a significant role in the overall performance and reliability of these advanced packaging systems. The RDLs serve as essential pathways for electrical connectivity, linking the microchips with external systems. Understanding the behavior of these layers under thermal and mechanical stress is crucial to the design and development of high-performance FOWLP structures, which are increasingly becoming the standard in modern electronics due to their high density and efficient use of space. The fabrication process for these complex structures began with the deposition of the PI 0 dielectric layer onto the carrier substrate at a controlled temperature of 210 °C, a critical step that ensured the proper insulation and protection of the underlying components. Precise temperature control was essential because any deviations could lead to defects in the dielectric layer, impacting the overall performance of the package. The PI 0 layer served as the foundational layer, providing the necessary insulation and mechanical support for the subsequent RDLs and dielectric layers that would be added in the later stages of the process.

In the second stage of the fabrication process, the first redistribution layer (RDL 1) was fabricated. This layer formed the initial network of interconnections, establishing the primary pathways for electrical signals within the FOWLP structure. The process of fabricating the RDL involved precise lithography and metallization techniques, which needed to be executed with high accuracy to ensure the correct alignment and functionality of the interconnections. Once RDL 1 was successfully formed, the process moved to the third stage, where the dielectric layer PI 1 was deposited. This layer served to insulate RDL 1 from subsequent redistribution layers, preventing any electrical short circuits and ensuring the structural integrity of the entire package. The steps in stages two and three, specifically the fabrication of the RDLs and dielectric layers, were subsequently repeated twice to complete the formation of the additional layers, namely RDL 2, PI 2, RDL 3, and PI 3. Each repetition of this process was conducted with extreme precision, as the accuracy and performance of the final FOWLP structure depend heavily on the correct fabrication of each layer. The RDLs must be perfectly aligned, and the dielectric layers must provide effective insulation without introducing any mechanical stress or defects that could affect the warpage behavior of the package. By repeating these steps, the multilayered structure of the FOWLP was built, with each layer contributing to the overall electrical and mechanical performance of the system.

Moreover, the repeated fabrication process, involving multiple RDLs and dielectric layers, introduced additional complexity in terms of thermal management and mechanical stability. Each layer, especially the redistribution layers, contributed to the overall thermal expansion characteristics of the package, which in turn influenced the warpage behavior during thermal cycling. The Shadow Moiré technique was particularly useful in capturing these subtle deformations, providing detailed insights into how each layer affected the overall warpage of the structure. By correlating the experimental data with the FEA simulations, this study was able to refine the predictive models, ensuring that they accurately represent the real-world behavior of FOWLP structures under varying conditions. The combination of advanced fabrication techniques, precise measurement methods like Shadow Moiré, and sophisticated FEA simulations provides a comprehensive approach to understanding and mitigating warpage in FOWLP. This detailed understanding is critical for the development of reliable and efficient semiconductor packaging solutions that meet the demands of modern electronics, where performance, miniaturization, and reliability are paramount.

**Figure 8:**
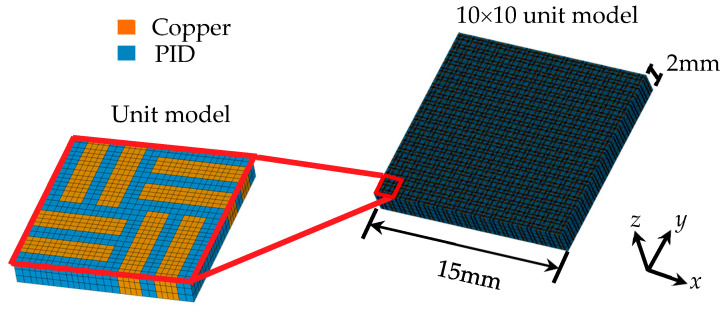
The model for the estimation of equivalent material properties.

Due to the inherent scale mismatch between the intricate and smaller features of the RDL pattern and the significantly larger 12-inch wafer, it became clear that utilizing a conventional direct modeling approach for FEA simulations is not practical. The vast difference in scale leads to computational challenges, as accurately representing every detail of the RDL pattern within the full-scale wafer model would result in an excessively large computational burden and unmanageable simulation times. Therefore, an FEA utilizing a detailed fine-mesh model can be directly applied to determine the effective orthotropic elastic properties of Cu circuit layers. This approach is particularly effective in capturing the critical parameters that influence these effective properties with high accuracy. However, it involves a highly intricate, time-intensive, and complex process for modeling and simulating the material behavior [27]. This methodology is succinctly referred to as the FEA-based effective approach [28]. The fundamental concept of this approach is to ensure that the elastic responses of the homogeneous equivalent continuum are in alignment with those of the original heterogeneous medium. Figure 8 depicts the model used in this study for the evaluation of equivalent material properties.

The effective CTEs of the Cu circuit layers could be simply calculated based on the strength of the materials:

(3) $$\alpha_{i}=\frac{\delta_{i}}{(\Delta T)L_{i}}\;(i=x,y,z)$$

where *δ_i_* is the thermal deformation, *α_i_* (*i* = *x*, *y*, *z*) stands for the effective CTE in the *i*th direction, Δ*T* denotes the temperature increment, and *L_i_* represents the side length of the Cu circuit layers in the *i*th direction.

In accordance with the generalized Hooke’s law, the stress–strain relationship of an orthotropic material is expressed as

(4) $$\varepsilon_{xx}=\frac{\sigma_{xx}}{E_{x}}+\frac{\upsilon_{xy}}{E_{y}}\sigma_{yy}+\frac{\upsilon_{zx}}{E_{z}}\sigma_{zz}$$

(5) $$\varepsilon_{yy}=\frac{\upsilon_{xy}}{E_{x}}\sigma_{xx}+\frac{\sigma_{yy}}{E_{y}}+\frac{\upsilon_{yz}}{E_{z}}\sigma_{zz}$$

(6) $$\varepsilon_{zz}=\frac{\upsilon_{zx}}{E_{x}}\sigma_{xx}+\frac{\upsilon_{yz}}{E_{y}}\sigma_{yy}+\frac{\sigma_{zz}}{E_{z}}$$

(7) $$\gamma_{yz}=\frac{\tau_{yz}}{G_{yz}}$$

(8) $$\gamma_{xz}=\frac{\tau_{xz}}{G_{xz}}$$

(9) $$\gamma_{xy}=\frac{\tau_{xy}}{G_{xy}}$$

where *ε*(*ε_x_*, *ε_y_*, *ε_z_*) and *σ*(*σ_x_*, *σ_y_*, *σ_z_*) are the normal strain and stress, respectively, *γ*(*γ_xy_*, *γ_yz_*, *γ_zx_*) and *τ*(*τ_xy_*, *τ_yz_*, *τ_zx_*) represent the shear strain and stress, respectively, and *υ*(*υ_xy_*, *υ_yz_*, *υ_zx_*) denotes Poisson’s ratio. In total, there are nine independent effective elastic constants to be determined for an orthotropic elastic material, which are *E_x_*, *E_y_*, *E_z_*, *υ_xy_*, *υ_yz_*, *υ_zx_*, *G_xy_*, *G_yz_*, and *G_xz_*. These constants can be simply derived based on Equations (4)–(9) through FEAs with a set of different loading and boundary conditions. The rest of the effective elastic constants, *υ_xy_*, *υ_yz_*, and *υ_zx_*, can be readily derived from the fact that the compliance matrix is symmetric.

Table 3 presents the material properties of the RDLs. The effective material properties were calculated based on the equations mentioned above. The Cu volume fraction was approximately 24.9% in RDL1, 35.7% in RDL2, 40.2% in RDL3, and 23.5% in RDL4. This effective material approach not only simplified the FEA simulation but also provided a more accurate representation of the mechanical behavior of the RDLs at the wafer scale. Without this approach, the simulation would either be too computationally expensive to be feasible or too simplified to yield meaningful results. By integrating the FEA-based effective approach, this study ensured that the essential mechanical properties of the RDLs were accurately captured, enabling precise predictions of warpage and other thermomechanical effects in the FOWLP structure. The combination of in-plane and out-of-plane CTE calculations allowed for a comprehensive understanding of the thermal behavior of the RDLs, contributing to the overall accuracy of the simulation. Ultimately, this approach underscores the importance of balancing computational efficiency with the need for accuracy in FEA simulations, particularly when dealing with complex multi-material systems like FOWLP. Through the use of effective material properties, this study was able to overcome the challenges posed by the scale mismatch between the RDL pattern and the 12-inch wafer, ensuring that the simulation results remained reliable and relevant to real-world applications in semiconductor packaging.

**Figure 9:**
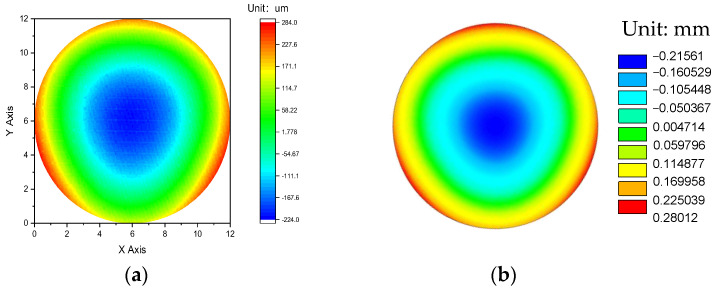
The (**a**) measured and (**b**) simulated warpage contour plots.

The warpage analysis results, obtained through both experimental measurements and FEA simulations, are presented in Figure 9 for a detailed comparison. These results are critical for understanding the complex thermomechanical behavior that occurs during the RDL fabrication process, a key stage in the production of FOWLP structures. Specifically, Figure 9a displays the experimentally measured warpage after the entire RDL fabrication process had been completed, showcasing the actual physical deformation of the structure. On the other hand, Figure 9b illustrates the corresponding warpage predicted by the FEA simulations, providing a theoretical representation of the same deformation based on the assumptions of the model and the material properties used in the simulation. Warpage refers to the differential deformation experienced by the structure along the *z*-axis, which is the vertical direction perpendicular to the wafer plane. Warpage is quantified as the difference between the maximum and minimum displacement values across the surface of the wafer, effectively measuring how much the structure bends or deforms due to thermal or mechanical stress. This deformation can be caused by several factors, including the differing CTEs between the various materials used in the packaging, as well as the internal stresses that develop during the RDL fabrication process.

The comparison between the experimental measurements and the simulated results revealed a high degree of correlation, which was crucial in validating the accuracy and reliability of the FEA model employed in this study. Such validation was essential for ensuring that the simulation can be trusted to predict real-world outcomes, which is particularly important when designing and optimizing semiconductor packaging technologies. According to the results, the experimentally measured warpage reached a value of 506.6 μm, while the simulated warpage value was 495.7 μm. This minimal deviation of only 10.9 μm between the experimental and simulated values demonstrates a remarkably close agreement, underscoring the precision of the FEA model used in this analysis. This small discrepancy between the measured and simulated warpage can be attributed to several factors, including slight variations in material properties, process conditions, or the inherent limitations of experimental measurement techniques. Nonetheless, the close alignment between these two values highlights the robustness and reliability of the FEA approach, confirming that the model is capable of accurately capturing the key physical phenomena that occur during the RDL fabrication process. The ability of the FEA model to predict warpage with such a high degree of accuracy is particularly noteworthy given the inclusion of the viscoelastic properties of the EMC in the simulation. Viscoelastic materials, such as the EMC, exhibit both elastic and time-dependent viscous behavior, making their mechanical response more complex than that of purely elastic materials. By accounting for these viscoelastic properties in the simulation, the model is able to more accurately reflect the real-world behavior of the FOWLP structure under thermal cycling conditions.

The close congruence between the experimental and simulated results serves as strong evidence of the accuracy and robustness of the proposed process modeling methodology for predicting warpage. This high level of agreement between the two sets of data not only validates the effectiveness of the FEA framework in capturing the complex thermomechanical behavior of the system but also emphasizes the capability of the model to provide reliable predictions of warpage under practical manufacturing conditions. In other words, the FEA-based approach proves to be a highly effective tool for understanding and predicting the deformation behavior of FOWLP structures during the critical RDL fabrication process, which is essential for optimizing these structures to minimize undesirable warpage. Moreover, the successful validation of the FEA model in this study paves the way for its future application in optimizing packaging designs to minimize thermally induced warpage in advanced electronic systems. By providing a reliable and accurate tool for predicting warpage, this methodology enables engineers and designers to test various design configurations and material choices in a virtual environment before implementing them in actual manufacturing processes. This ability to simulate and predict warpage behavior in advance allows for more efficient and cost-effective design optimization, reducing the risk of failures and defects in the final product. Furthermore, the accuracy of the FEA model, as thoroughly validated through the comparison with experimental data, lays a strong foundation for the development of an AI-based predictive platform for warpage analysis. The data derived from the FEA simulations, having been confirmed to be accurate and reliable, will serve as a highly credible and robust dataset for training and validating the AI model. The integration of FEA-derived data into the AI platform will significantly enhance the precision and reliability of the AI predictions, ensuring that the platform can provide accurate and trustworthy results in real-world applications.

The use of AI in warpage prediction offers several advantages, including the ability to quickly analyze large datasets and identify complex patterns that may not be immediately apparent through traditional methods. By leveraging machine learning algorithms, the AI platform can learn from the FEA data and improve its predictive capabilities over time, becoming more accurate and efficient with each iteration. This integration of FEA and AI will facilitate the more effective optimization and control of warpage in future packaging technologies, allowing for faster and more reliable design iterations. In practical terms, this AI-based predictive platform could be used to simulate the effects of various design parameters, such as material properties, layer thicknesses, and process conditions, on the warpage behavior of FOWLP structures. By providing accurate predictions of how these factors will influence warpage, the platform will enable engineers to make informed decisions during the design phase, ultimately leading to more robust and reliable packaging solutions. This will be particularly valuable in the development of next-generation electronic systems, where the demand for smaller, faster, and more efficient devices requires increasingly complex packaging designs with tight tolerances for warpage and other mechanical deformations. In conclusion, the results of this study firmly establish the credibility and effectiveness of the FEA-based approach in predicting warpage phenomena during the RDL fabrication process. The close agreement between the experimental measurements and the FEA simulations underscores the accuracy and robustness of the model, while the inclusion of viscoelastic properties further enhances its predictive capabilities. The successful validation of the FEA model not only demonstrates its usefulness in current packaging design optimization efforts but also lays the groundwork for the development of an AI-based predictive platform, which will enable even more efficient and accurate warpage analysis in the future. Through this integration of FEA and AI, this study contributes to the ongoing advancement of semiconductor packaging technologies, helping to meet the ever-growing demands of the electronics industry for high-performance, reliable, and miniaturized devices.

### 5.2. Establishment of Training/Test and Validation Datasets

**Figure 10:**
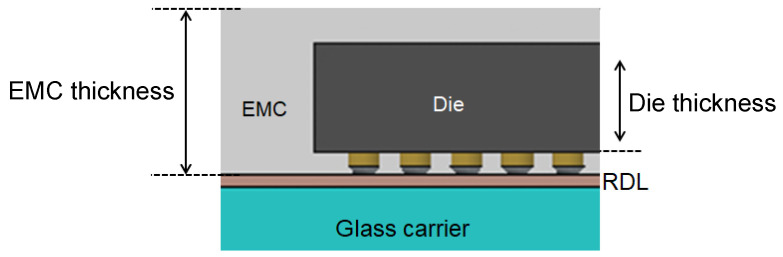
Specifications of the die thickness and EMC thickness.

In this study, five critical parameters were meticulously identified as the most influential factors contributing to thermal stress-induced warpage in FOWLP structures. These parameters include the Die/Package area ratio, the die thickness, the EMC thickness, and two key material properties of the EMC: Young’s modulus and the CTE. Figure 10 provides detailed specifications regarding the die thickness and EMC thickness. Each of these parameters was selected for its substantial impact on the thermomechanical performance of FOWLP structures, particularly under thermal cycling, a prevalent operational stressor in advanced packaging technologies that significantly influences warpage behavior.

To further understand how changes in these five parameters affect warpage, they were systematically incorporated into the development of an AI model designed to predict warpage behavior induced by thermal stress. The AI model, built using FEA-generated data, enables engineers to optimize FOWLP structures by minimizing warpage. The training dataset, consisting of 1200 data points, was created through FEA simulations, each representing a unique combination of the five parameters and corresponding warpage behavior, as detailed in Table 4. This comprehensive dataset offers a solid foundation for the AI model to accurately predict warpage across various design configurations. Additionally, this study systematically investigated the effects of the Die/Package area ratio, die thickness, and EMC thickness, as well as Young’s modulus and the CTE of the EMC, on warpage behavior. The Die/Package ratio was varied across 12 distinct percentage values ranging from 10% to 60% to capture a broad spectrum of design possibilities. Similarly, realistic variations in the die and EMC thicknesses were examined, as these parameters critically influence the structural integrity and thermal stress response of the package. The mechanical properties of the EMC were evaluated across a range of Young’s modulus values (5, 10, 15, 20, and 25 GPa) and CTE values (5, 7, 10, and 15 ppm), reflecting different material behaviors under thermal stress. By exploring these variations, the study provides insights into the stiffness and thermal expansion behavior of the EMC, which are critical in minimizing warpage. Through the comprehensive simulation process, the generated dataset offers a robust foundation for in-depth analysis and accurate predictions across a wide variety of design configurations. Ultimately, the findings from this study contribute to a deeper understanding of the factors that influence warpage in FOWLP structures and provide valuable guidance for future packaging design, enhancing the reliability and performance of these technologies.

### 5.3. A Comparison of the Prediction Results from Different Learning Models

The SVR model was initialized with a radial basis function (RBF) kernel, commonly used to handle non-linear relationships in data. The parameter C, which is set to 100, controls the regularization of the model, with a higher value leading to a smaller margin and less tolerance for misclassification, making it more sensitive to training data. The gamma parameter, set to 0.1, defines the influence range of a training point, where a lower gamma extends the influence farther, and a higher gamma restricts it to nearby points. The epsilon parameter, set to 0.1, specifies the margin of tolerance within which no penalty is applied to errors in the training data. The model is trained on standardized input data, allowing it to make continuous predictions based on normalized features. In the RF model, the n_estimators parameter, set to 100, determines the number of decision trees used in the ensemble. With 100 trees, the model gains robustness and stability by averaging the outputs of multiple trees, reducing overfitting. The random_state parameter is set to 42, ensuring reproducibility by fixing the randomness in the internal processes of the algorithm. These parameters help balance randomness and repeatability while maximizing the predictive power of the model.

The GBR model uses 100 estimators, performing 100 boosting iterations to refine predictions and reduce errors. The learning rate is set to 0.1, controlling the contribution of each tree to the final prediction. The maximum depth of the trees is limited to 3, preventing overfitting, and the random_state parameter is set to 0 for reproducibility. Together, these parameters guide the model in handling bias, variance, and the overall accuracy. For the KNN model, n_neighbors = 3 specifies that the three closest neighbors will be used for regression. Both the input data and the target variable are normalized during training, and predictions are denormalized back to their original scale after the model completes the prediction process. The KRR model employs an RBF kernel, which captures non-linear relationships by measuring data point similarities based on their distance in the feature space. The alpha parameter, set to 1.0, controls the regularization strength, balancing the trade-off between fitting the training data and generalizing to unseen data. A higher alpha increases regularization to reduce overfitting, while a lower alpha allows the model to capture more intricate patterns, potentially at the cost of overfitting. This combination of the RBF kernel and regularization through the value of alpha enables KRR to handle non-linear regression tasks effectively while managing the risk of overfitting.

The input layer of the RNN model accepts a shape of (5, 1), indicating that each sample has five time steps, with each step containing a single feature. In the RNN and LSTM layers, 128 units are used, meaning they maintain 128 hidden units. Both layers use the ReLU activation function, which introduces non-linearity into the model to help it learn complex patterns. The final output layer, named ‘output1’, is a single-unit Dense layer, reflecting that the model predicts a single target variable, such as the warpage value. In the RNN model, the SimpleRNNCell, with its 128 units, maintains the hidden state, while in the LSTM model, the LSTM layer processes sequences of data across multiple time steps. Both models are compiled using the Adam optimizer with a learning rate of 0.001, which is suited for gradient-based optimization. The loss function used is the MSE, appropriate for regression tasks as it measures the squared differences between predicted and actual values.

Similarly, in the GRU model, the main parameter is the number of units, set to 128, which defines the dimensionality of the output space. The GRU is wrapped in a Bidirectional layer, which processes the input sequence both forward and backward, allowing the model to capture information from past and future time steps. The MLP model was designed with an input layer that accepts five features. It has three Dense layers, each containing 128 neurons and all using the ReLU activation function. The final output layer is a single neuron for prediction. This model is also compiled using the Adam optimizer with a learning rate of 0.001, and the MSE is used as the loss function. In all these models, normalization is applied to the input data, scaling them based on the mean and standard deviation, ensuring that all features contribute equally. Similarly, the output variable is normalized before training, and the predicted values are denormalized after prediction for better interpretability. All models are trained with 10,000 epochs and a batch size of 240, with verbosity turned off during training to minimize the console output.

In this study, a total of 10 sets of FOWLP dimensions and material parameters were meticulously selected to enable a thorough and comprehensive comparison between the performance of various machine learning models and deep learning models. The objective of this analysis was to evaluate the predictive accuracy and reliability of these models in estimating warpage, a critical factor in FOWLP applications, by comparing their predictions to those obtained from FEM simulations, which served as a benchmark for assessing the validity of the predictions. The selection of 10 distinct sets of parameters ensured that a broad range of conditions and scenarios was represented, thereby allowing for a robust evaluation of the models’ generalizability and performance across different configurations.

Table 5 provides an in-depth comparison between the FEM results and the corresponding predictions generated by five distinct machine learning models. These models were chosen for their widespread use and applicability in regression tasks, making them ideal candidates for evaluating their effectiveness in this particular domain. The models compared include SVR, the RF model, GBR, the KNN, and KRR. Each model was trained and tested on the selected dataset, and the results were evaluated based on the average deviation and standard deviation from the FEM results, which served as the reference values for warpage predictions. The analysis revealed the following deviations for each machine learning model when compared to the FEM results: SVR exhibited an average deviation of 18.5% with a standard deviation of 15.4%, the RF model demonstrated a deviation of 6.2% with a standard deviation of 6.2%, GBR recorded a deviation of 21.6% with a standard deviation of 26.5%, the KNN showed a deviation of 14.9% with a standard deviation of 19.8%, and KRR presented a deviation of 17.3% with a standard deviation of 16.4%. These values offer critical insights into the relative performance of each model, highlighting the strengths and limitations of different machine learning approaches when applied to warpage prediction tasks in FOWLP. The lower the deviation, the more closely the predictions of the model aligned with the FEM results, indicating higher accuracy and reliability. It is evident from the results that the RF model exhibited the smallest deviation from the FEM results, with an average deviation of 6.2%, and this deviation was notably lower in comparison to that of the other machine learning models included in the analysis. The relatively low standard deviation associated with the RF model also indicates a higher level of consistency across different test cases, further underscoring its reliability. This suggests that, among the models evaluated, the RF model demonstrates a superior predictive capability and a higher level of accuracy in estimating warpage for FOWLP. The ensemble nature of the random forest algorithm, which aggregates the outputs of multiple decision trees, likely contributes to its robustness and ability to generalize across various parameter sets. The performance of this model highlights its suitability for regression tasks in this area, offering both accuracy and stability, making it a strong candidate for predictive applications in the field of FOWLP.

In contrast, Table 6 presents a similarly comprehensive comparison between the predictive results generated by various deep learning models and the corresponding FEM results. Unlike traditional machine learning models, deep learning models are particularly well suited for handling complex, high-dimensional datasets and capturing non-linear relationships within the data. The deep learning models compared in this study included RNN, GRU, MLP, and LSTM models. These models are known for their ability to model sequential data and learn complex patterns over time, which makes them ideal for this task, where warpage predictions depend on multiple interacting factors. A detailed analysis of the data revealed the average deviation and standard deviation for each deep learning model when compared to the FEM results. Specifically, the RNN model demonstrated a remarkably low deviation of 0.21% with a standard deviation of 0.23%, indicating a high level of accuracy and consistency in its predictions. The GRU model exhibited a deviation of 0.55% with a standard deviation of 0.56%, while the MLP model recorded a deviation of 0.60% with a standard deviation of 0.68%. The LSTM model, another popular variant of recurrent neural networks, showed a deviation of 0.34% with a standard deviation of 0.35%. The comparison clearly indicates that the predictions generated by all deep learning models significantly outperformed those of the machine learning models, including the most accurate machine learning model, the RF model, in terms of both accuracy and precision. This stark contrast between the results highlights the superior capability of deep learning models to capture complex patterns, interactions, and relationships within the data, offering a level of predictive performance that traditional machine learning models cannot match.

The ability of deep learning models to process and learn from high-dimensional data without extensive feature engineering, combined with their capacity to model non-linear relationships, makes them particularly effective for tasks involving complex, multi-factorial phenomena such as warpage prediction in FOWLP. The substantial improvement in accuracy provided by the deep learning models is evident in the significant reduction in both the average deviation and the standard deviation across all models when compared to the FEM results. This improvement underscores the potential of deep learning models for more reliable and precise predictions in engineering applications, where accuracy is paramount for ensuring optimal performance and design integrity. The ability of deep learning models to consistently produce high-quality predictions across different parameter sets further reinforces their suitability for tasks involving complex systems and processes. Furthermore, within the set of deep learning models evaluated, the RNN model stands out as the top performer, demonstrating the smallest average deviation and standard deviation. This finding suggests that the RNN model not only produces predictions that are closest to the FEM results but also maintains a high level of consistency across different test cases, further solidifying its position as the most reliable model in this study. The ability of the RNN model to capture the sequential dependencies and interactions between different parameters likely contributes to its superior performance, making it an ideal candidate for warpage prediction in FOWLP applications. Consequently, based on these findings, this study concluded that the RNN deep learning model would be adopted as the core algorithm for the AI-based prediction platform. Its demonstrated ability to provide the most precise and consistent predictions, in comparison to both machine learning and other deep learning models, reinforced its suitability for integration into this predictive framework. The adoption of the RNN model was expected to enhance the reliability and effectiveness of the platform in future applications, offering more accurate and reliable warpage predictions, which are crucial for optimizing FOWLP designs and ensuring the success of advanced packaging technologies.

### 5.4. An AI Prediction Platform with a Graphical User Interface (GUI)

This study introduces a sophisticated AI prediction platform that operates via a uniform resource locator (URL) and features a comprehensive GUI, designed to streamline the user interaction process with complex AI models. The development of this platform addresses the growing need for accessible yet powerful AI-driven predictive tools, enabling users to engage with advanced machine learning algorithms in an intuitive and efficient manner. The entire framework of the platform, from its architectural design to its predictive functionality, was meticulously constructed using the Python programming language v3.11. The extensive library support, flexibility, and robust machine learning frameworks provided by Python made it the ideal choice for developing such a platform, allowing for the integration of complex models and ensuring high computational efficiency. The activation of this AI prediction platform through a URL necessitated the establishment of a reliable host server port, which acts as the gateway for communication between the user interface and the backend AI models. The server port plays a critical role in ensuring seamless data flow, allowing users to submit inputs and retrieve predictions without requiring direct access to the local machine hosting the models. However, to facilitate external network access to this host server port, it became imperative to configure a virtual server. The virtual server functions as an intermediary, connecting external users to the host server by providing a secure and stable channel for data transmission. Without this configuration, the platform would remain inaccessible to external networks, limiting its utility to local use only.

The process of configuring a virtual server introduces several key technical challenges that must be addressed to ensure the effectiveness of the platform. One of the primary concerns in this context is network security. Since the platform is accessible via the internet, robust security measures must be implemented to prevent unauthorized access, protect sensitive data, and safeguard the AI models from potential cyber threats. These security measures typically include the implementation of encrypted communication protocols such as HTTPS, advanced firewall configurations, and multilayered user authentication systems. By integrating these security mechanisms, the platform is protected from malicious attacks, ensuring that only authorized users have access to its predictive capabilities. Beyond security considerations, the performance optimization of the virtual server is another crucial aspect of the design of the platform. The AI models incorporated into the system are computationally intensive, particularly when they process large datasets or perform sophisticated algorithmic operations. As such, the virtual server must be provisioned with adequate computational resources, including sufficient CPU power, memory allocation, and storage capacity, to ensure that the platform can handle high-volume requests and deliver rapid predictions. In addition, attention must be given to the network latency and bandwidth management, as these factors directly affect the responsiveness of the platform. A high degree of optimization will ensure that the platform provides real-time feedback and remains responsive even when accessed from remote locations or under heavy user loads.

Once the virtual server has been fully configured and integrated with the host server port, the AI prediction platform will become operational, accessible through a simple URL. This web-based access eliminates the need for complex local installations or intricate technical setups, significantly reducing the barrier to entry for users who may not have extensive technical knowledge. The URL serves as the interface through which users can interact with the platform, launching the GUI that facilitates access to the underlying AI models. The GUI itself is designed to prioritize the user experience, offering a streamlined and intuitive interface that allows for easy navigation through the various features and functions of the system. The GUI provides users with the capability to input data, adjust model parameters, and visualize results in a coherent and organized manner. This interface is not merely a superficial layer but plays an integral role in enhancing the ability of users to interact with the AI models effectively. The dynamic nature of the GUI allows users to modify input variables, immediately observe how these changes impact model predictions, and fine-tune parameters to better understand the underlying relationships captured by the AI models. Such interactivity promotes a deeper understanding of the AI decision-making process, facilitating more informed decision-making based on the model outputs. The visualization components embedded within the GUI are carefully designed to translate complex prediction data into easily interpretable formats, using charts, graphs, and other graphical tools to represent the model outputs.

From a technical standpoint, the process of developing this AI prediction platform involved multiple stages, each requiring significant attention to detail. Establishing the host server port was the initial step, providing the foundational infrastructure necessary for communication between the user interface and the AI models. However, to make this infrastructure accessible externally, the creation of a virtual server was essential. This required configuring the virtual server to ensure that it could securely and efficiently handle the data requests being transmitted via the URL. In addition to setting up the server architecture, the development of the GUI was a critical component in ensuring the overall usability of the platform. The GUI was designed to abstract the complexity of the AI models, presenting the user with a clear, interactive environment that simplifies the predictive process while maintaining the computational rigor of the system. The entire system architecture reflects a balance between accessibility and complexity. The platform leverages the power of Python machine learning libraries to deliver sophisticated predictive capabilities, while the GUI ensures that these capabilities are presented in an accessible manner, allowing users of varying technical expertise to engage with the AI models effectively. This fusion of advanced machine learning technology with user-centric design underscores the utility of the platform as both a powerful predictive tool and an accessible application for practical use.

**Figure 11:**
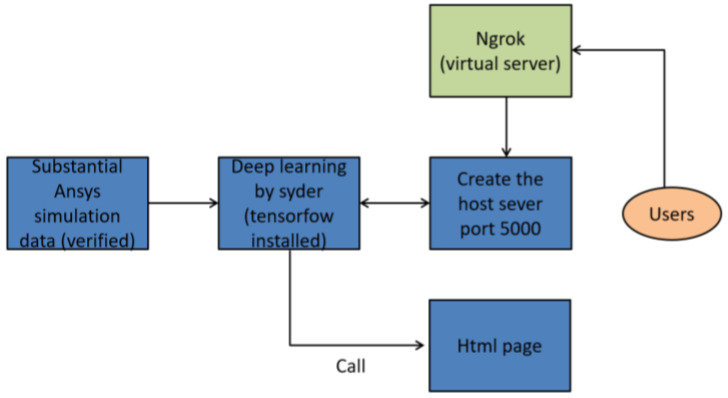
The process of constructing a GUI-based AI prediction platform that operates through a URL involves several technical stages.

Figure 11 offers a detailed depiction of the workflow involved in establishing the GUI-based AI prediction platform that operates via a URL. It outlines the sequential steps required to set up the host server port, configure the virtual server, and ensure secure and efficient communication between the user interface and the backend AI models. The figure also highlights the interaction between the virtual server and external users, illustrating how the system facilitates real-time predictions and dynamic feedback through the GUI. This visual representation serves as an essential guide for understanding the technical infrastructure that supports the operation of the platform, providing additional insights into the underlying processes that enable its seamless functionality. By enabling web-based access to complex AI models, this platform represents a significant advancement in the field of predictive analytics, offering users the ability to harness the power of machine learning algorithms in a practical and efficient manner. The design of the platform, which integrates advanced security measures, computational optimization, and an intuitive GUI, ensures that it is both robust and user-friendly. This balance between technical complexity and ease of use makes the platform an ideal tool for a wide range of applications, from academic research to industrial use, providing a flexible solution for AI-driven predictive analysis.

The integration of a virtual server with the host server port allows for a scalable and secure platform that can accommodate multiple users and varying computational demands. This scalability ensures that the platform can grow in response to increased usage, maintaining its performance and reliability as it scales. Additionally, the focus on security protocols guarantees that the platform will remain protected against unauthorized access and potential vulnerabilities, ensuring that it can be trusted for use in sensitive applications where data security is paramount. The development of this platform marks a step forward in making sophisticated AI technologies more accessible to users across different domains, democratizing access to predictive analytics tools.

**Figure 12:**
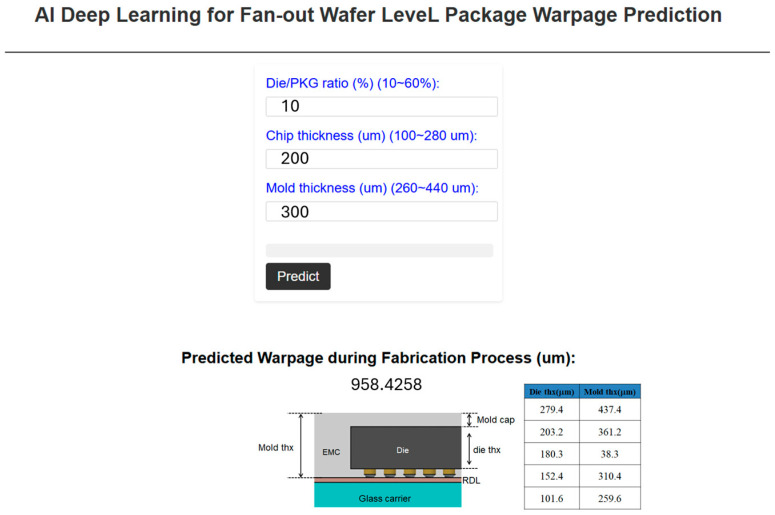
The interface of the GUI-based AI prediction platform.

Figure 12 provides a detailed illustration of the AI prediction platform, equipped with a GUI that operates through a URL-based system. This platform allows users to interact with complex AI-driven predictions in a streamlined and efficient manner. By entering specific dimensional parameters, users can initiate the predictive process by clicking the “predict” button, immediately receiving warpage values for the packaging structures under consideration. This instant feedback mechanism is designed to facilitate rapid and accurate decision-making, providing engineers and designers with a reliable tool to guide their structural designs. The platform thus serves as a valuable resource for optimizing design parameters and improving the overall performance of packaging structures. By automating the predictive process, the platform reduces the manual workload typically associated with traditional modeling and simulation techniques, allowing users to obtain results in a fraction of the time.

The ability of the system to operate via a URL adds significant value by enabling remote access to the platform from any internet-connected device. This design feature ensures that users can benefit from the AI capabilities of the platform regardless of their physical location, thereby enhancing accessibility and user convenience. The platform is not only capable of delivering fast predictions but also ensures a high degree of accuracy, given its reliance on advanced AI algorithms that have been rigorously trained to model warpage behavior under various conditions. This capability offers a robust framework for practical applications in real-world scenarios, making it a critical tool for professionals who require precise predictions in their design workflows.

However, recognizing the limitations that may arise from network connectivity issues, the platform also includes a standalone version of the GUI-based AI prediction tool. For users whose computational environments lack reliable internet access, the offline version provides an equally powerful solution. The standalone version maintains the same core functionality and predictive accuracy as the URL-based system but is tailored for local use, ensuring uninterrupted operation without the need for external server connections. This adaptability is particularly important for users operating in restricted environments, such as laboratories or industrial settings where network access may be limited or unavailable. In the subsequent sections, an in-depth examination of the standalone version of the GUI-based AI prediction platform will be presented. This version retains all of the advanced features of the online platform, including its user-friendly interface and the precise predictive capabilities powered by machine learning algorithms. However, the standalone application has been optimized to run on local machines, providing users with a high-performance tool that does not rely on cloud infrastructure. By ensuring that the platform is fully functional in both online and offline modes, the system demonstrates its versatility and wide applicability across various fields, from academic research to industrial engineering. The flexibility offered by the platform makes it a critical asset for improving design efficiency and enhancing the predictive accuracy of packaging structure analyses, regardless of the working environment of the user.

**Figure 13:**
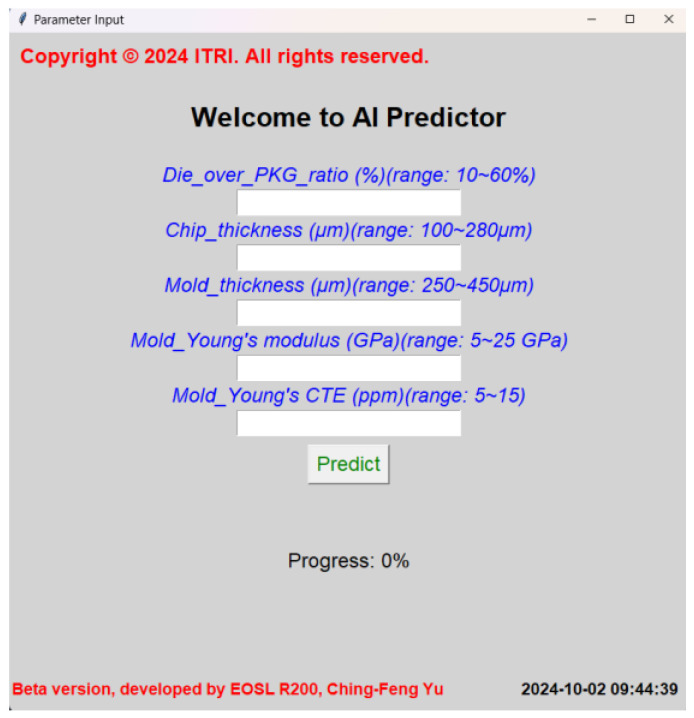
The standalone version of the AI prediction platform with a GUI.

The process of establishing the standalone version of the GUI-based AI prediction platform was notably simpler when compared to the method used for the URL-based platform. This streamlined approach involved embedding the GUI directly into the program code and subsequently converting it into an executable file (.exe). Once the executable file is generated, users can easily run the GUI-based AI prediction platform by merely clicking on the file, bypassing the need for external server configurations or network access. This localized approach ensures that the platform can function seamlessly on individual machines without requiring an internet connection, thus providing users with enhanced accessibility and convenience in environments where network connectivity might be unreliable or unavailable. When the platform is launched, users are greeted by a welcome message, which can be customized based on specific needs or user preferences. The main interface of the platform, as illustrated in Figure 13, maintains a clean and intuitive layout, allowing users to engage with the prediction model in a straightforward manner. The interface design follows the same operational logic as the URL-based version of the platform, ensuring consistency in the user experience across both versions.

Once the platform is fully opened, users are prompted to input dimensional parameters into the designated fields. These parameters, which are critical to determining the warpage behavior of the packaging structures, can be easily entered by the user. After the relevant values have been provided, the user simply clicks the “predict” button to initiate the AI-driven prediction process. The platform quickly processes the input data and delivers accurate warpage values, offering a comprehensive prediction of the structural behavior. This immediate feedback is crucial for design engineers, as it provides them with a quick and reliable tool for assessing potential design outcomes. The warpage prediction helps inform key design decisions, ensuring that packaging structures are optimized for both performance and durability. Furthermore, the operational simplicity of the standalone version of the platform enhances its utility in a wide range of applications. With no need for complex server setups or network configurations, users can deploy the platform in diverse working environments, from individual workstations in research labs to industrial settings where network access may be restricted. The standalone nature of the executable file also ensures that the platform is portable, enabling users to move it between different machines or share it with colleagues without the need for additional installations or configurations. The consistency between the standalone and URL-based versions of the platform ensures that users transitioning between the two will encounter a familiar interface and workflow. Both versions allow users to input dimensional parameters, initiate predictions with the click of a button, and receive real-time feedback on the warpage behavior of the packaging structures. This uniformity in operation promotes ease of use, as users are not required to learn new systems or interfaces when switching between the standalone and web-based platforms. The prediction results generated by both versions are equally accurate, thanks to the underlying AI models that power the platform, ensuring that the reliability and precision of the predictions remain unchanged regardless of the deployment method.

In essence, the standalone version of the GUI-based AI prediction platform offers a versatile and practical solution for users who require advanced predictive capabilities in environments where network connectivity may be limited or nonexistent. Its straightforward installation and operational process, combined with its powerful AI-driven prediction capabilities, make it an essential tool for design engineers and researchers alike. By providing quick and reliable warpage predictions, the platform supports informed decision-making and contributes to the overall efficiency and effectiveness of the design process. Through the localized execution of the platform, users gain access to a robust predictive tool without the need for complex setups, making it an invaluable resource for professionals working in a variety of fields. The ability of the platform to deliver consistent results across both standalone and URL-based versions ensures that it remains a highly flexible solution, capable of adapting to the specific needs and constraints of different working environments. In this way, the standalone GUI-based AI prediction platform significantly enhances the accessibility and utility of advanced AI technologies in the design and analysis of packaging structures.

## 6. Conclusions

In this study, the development of a GUI-driven AI prediction platform for FOWLP warpage behavior prediction was comprehensively explored. This platform integrates FEA with advanced AI techniques to provide highly accurate, real-time predictions of thermal stress-induced warpage in FOWLP structures. By incorporating both URL-based and standalone versions, the system offers flexibility and accessibility to engineers, allowing them to interact with complex predictive models without requiring extensive programming knowledge. One of the major innovations of this platform is the seamless user interface, which allows users to input critical design parameters such as the die-to-package area ratio, die thickness, and the properties of the EMC into the AI model. This feature effectively simplifies the traditionally complex process of packaging design and simulation, making advanced modeling techniques more accessible to a broader range of users, particularly those without specialized expertise in these areas.

A key strength of the system lies in its ability to generate real-time, high-precision warpage predictions. This capability allows designers to rapidly evaluate different design variations, significantly reducing the need for time-consuming experimental methods. It also offers an efficient alternative to traditional FEA-based approaches, which can be computationally intensive and slow. The AI prediction tool integrates a wide range of machine learning models such as GBR, SVR, the KNN, KRR, and the RF model. In addition to these machine learning approaches, it employs deep learning models including an RNN, GRU, MLP, and LSTM network. These models have been rigorously trained using FEA-generated data to ensure they achieve high levels of predictive accuracy and reliability in practical applications.

The comparison of the predictive performance of these models revealed that deep learning techniques, particularly the RNN, significantly outperform traditional machine learning models in terms of both accuracy and consistency. As demonstrated in Table 6, the RNN model exhibited the smallest deviation from the FEA results, with an average deviation of only 0.21% ± 0.23%. This marked improvement in accuracy makes the RNN model the most effective option for predicting warpage behavior in FOWLP structures. Given these results, the RNN model was selected as the core algorithm for the AI prediction platform, ensuring that users benefit from the highest level of precision in real-time warpage predictions.

The integration of the high-performing RNN model into the GUI-driven platform provides engineers with a powerful, user-friendly tool for optimizing electronic packaging designs more effectively. This AI-based approach minimizes the risks associated with thermal warpage, which can lead to performance degradation or failure in next-generation electronic devices. By enabling real-time feedback and a streamlined simulation process, the platform enhances design workflows, allowing engineers to test multiple configurations and make data-driven decisions quickly. This represents a significant advancement in the field of electronic packaging, supporting the development of smaller, more reliable, and more efficient packaging solutions for cutting-edge technologies.

## Figures and Tables

**Table 1:** Material properties of each component [6].

Material	Young’s Modulus (GPa)	Poisson’s Ratio	Coefficient of Thermal Expansion (CTE)
Si	131	0.26	2.8
PI	3.3	0.3	52.5
Cu	120	0.4	17.5
Glass carrier	70.9	0.29	5

**Table 2:** Fitted Prony series coefficients.

*i*	*τ_i_*	*g_i_*	*i*	*τ_i_*	*g_i_*
1	1.0 × 10^19^	2.33 × 10^−14^	11	1.0 × 10^9^	0.1209
2	1.0 × 10^18^	2.33 × 10^−14^	12	1.0 × 10^8^	0.09685
3	1.0 × 10^17^	2.33 × 10^−14^	13	1.0 × 10^7^	0.07191
4	1.0 × 10^16^	2.33 × 10^−14^	14	1.0 × 10^6^	0.06336
5	1.0 × 10^15^	2.33 × 10^−14^	15	1.0 × 10^5^	0.05796
6	1.0 × 10^14^	2.40 × 10^−14^	16	1.0 × 10^4^	0.0473
7	1.0 × 10^13^	1.86 × 10^−12^	17	1.0 × 10^3^	0.03086
8	1.0 × 10^12^	0.01558	18	1.0 × 10^2^	0.04494
9	1.0 × 10^11^	0.109	19	1.0 × 10^1^	0.01368
10	1.0 × 10^10^	0.1342	20	1.0 × 10^0^	0.07303

**Table 3:** Material properties of the RDLs.

		Young’s Modulus (GPa)			Poisson’s Ratio			Shear Modulus (GPa)			CTE (ppm/°C)		
Mat.	Cu Content	$E_{x}$	$E_{y}$	$E_{z}$	$\nu_{xy}$	$\nu_{yz}$	$\nu_{xz}$	$G_{xy}$	$G_{yz}$	$G_{xz}$	$\alpha_{x}$	$\alpha_{y}$	$\alpha_{z}$
RDL4	23.5%	3.93 × 10^−10^	3.93 × 10^−10^	25.85	0.12	1.79 × 10^−12^	1.79 × 10^−12^	4 × 10^−11^	9.64	9.64	5.87	5.87	0.17
RDL3	40.2%	14.0	14.0	46.9	0.32	0.096	0.096	5.72	17.54	17.54	36.11	36.11	18.81
RDL2	35.7%	12.8	12.8	42.2	0.32	0.097	0.097	5.31	15.77	15.77	37.93	37.93	19.16
RDL1	24.9%	10.5	10.5	30.8	0.316	0.108	0.108	4.39	11.52	11.52	41.81	41.81	20.48

**Table 4:** Training data from FEA with 1200 data points for five input features.

Feature Name	Level
Die/Package (%)	10, 15, 20, 25, 30, 35, 40, 45, 48, 50, 55, 60
Die thickness (μm)	101.6, 152.4, 180.3, 203.2, 279.4
EMC thickness (μm)	256.6, 307.4, 335.3, 358.2, 434.4
EMC Young’s modulus (GPa)	5, 10, 15, 20, 25
EMC CTE (ppm)	5, 7, 10, 15

**Table 5:** Comparison between FEM results and predicted values from machine learning approaches.

Design	Die/PKG (%)	Die Thickness (μm)	Mold Thickness (μm)	Mold E (GPa)	Mold CTE (CTE)	Warpage FEM (μm)	SVR	RF	GBR	KNN	KRR
1	10	203.2	358.2	25	5	570.6	493.1	557.6	526.1	937.9	707.0
2	15	180.3	335.3	15	5	266.6	214.2	251.8	272.6	276.9	332.23
3	20	152.4	307.4	20	15	4702.1	4762.0	4720.5	4863.1	4713.6	4958.4
4	25	101.6	256.6	10	7	560.6	516.1	476.8	403.4	563.7	472.8
5	30	101.6	256.6	25	15	4550.6	4473.3	4491.5	4678.4	4556.0	4331.5
6	35	152.4	307.4	5	15	698.9	573.0	711.6	685.8	607.5	669.3
7	48	152.4	307.4	10	15	906.9	805.8	894.6	924.9	1210.6	743.7
8	50	180.3	335.3	10	10	101.5	209.7	115.1	363.5	148.0	226.7
9	55	101.6	256.6	10	7	215.8	180.7	246.7	451.0	213.8	278.1
10	60	203.2	358.2	15	10	271.8	218.1	280.3	334.2	172.7	281.0

**Table 6:** Comparison between FEM results and predicted values from deep learning approaches.

Design	Die/PKG (%)	Die Thickness (μm)	Mold Thickness (μm)	Mold E (GPa)	Mold CTE (CTE)	Warpage FEM (μm)	RNN	GRU	MLP	LSTM
1	10	203.2	358.2	25	5	570.6	571.3	568.0	572.4	568.3
2	15	180.3	335.3	15	5	266.6	266.7	263.1	266.4	269.7
3	20	152.4	307.4	20	15	4702.1	4703.0	4700.3	4711.1	4698.4
4	25	101.6	256.6	10	7	560.6	558.5	556.7	557.8	557.0
5	30	101.6	256.6	25	15	4550.6	4551.5	4546.7	4558.8	4546.1
6	35	152.4	307.4	5	15	698.9	699.3	698.9	696.4	697.5
7	48	152.4	307.4	10	15	906.9	906.3	905.8	906.5	906.1
8	50	180.3	335.3	10	10	101.5	100.8	100.6	99.6	101.5
9	55	101.6	256.6	10	7	215.8	216.3	219.2	212.1	215.3
10	60	203.2	358.2	15	10	271.8	273.0	271.0	270.0	273.2

## Data Availability

The original contributions presented in this study are included in the article. Further inquiries can be directed to the corresponding author.

## References

[1] Waldrop M.M. (2016). The chips are down for Moore’s law. Nature.

[2] Lau J.H. (2022). Recent advances and trends in advanced packaging. IEEE Trans. Compon. Packag. Manuf. Technol..

[3] Huang Y.W., Chiang K.N. (2022). Study of shear locking effect on 3D solder joint reliability analysis. J. Mech..

[4] Zhao J., Chen Z., Qin F., Yu D. (2022). Thermo-mechanical reliability study of through glass vias in 3D interconnection. Micromachines.

[5] Liu W.W., Weng B., Li J., Yeh C.K. (2019). FCCSP IMC growth under reliability stress following automotive standards. J. Microelectron. Electron. Packag..

[6] Yu C.F., Huang Y.W., Ouyang T.Y., Cheng S.F., Chang H.H., Hsiao C.C. (2022). Suppression strategy for process-induced warpage of novel fan-out wafer level packaging. Microelectron. Reliab..

[7] Chen C., Su M., Ma R., Zhou Y., Li J., Cao L. (2022). Investigation of warpage for multi-die fan-out wafer-level packaging process. Materials.

[8] Van Dijk M., Huber S., Stegmaier A., Walter H., Wittler O., Schneider-Ramelow M. (2022). Experimental and simulative study of warpage behavior for fan-out wafer-level packaging. Microelectron. Reliab..

[9] Cheng H.C., Wu Z.-D., Liu Y.C. (2020). Viscoelastic warpage modeling of fan-out wafer-level packaging during wafer-level mold cure process. IEEE Trans. Compon. Packag. Manuf. Technol..

[10] Lee C.C., Chang C.P., Huang P.C. (2023). Development and demonstration on process-oriented warpage simulation methodology of fan-out panel-level package in multilevel integration. IEEE Trans. Compon. Packag. Manuf. Technol..

[11] Kavitha S., Varuna S., Ramya R. A comparative analysis on linear regression and support vector regression. Proceedings of the 2016 Online International Conference on Green Engineering and Technologies (IC-GET).

[12] Hsiao H.Y., Chiang K.N. (2021). AI-assisted reliability life prediction model for wafer-level packaging using the random forest method. J. Mech..

[13] Praveena M., Jaiganesh V. (2017). A literature review on supervised machine learning algorithms and boosting process. Int. J. Comput. Appl..

[14] Ghawi R., Pfeffer J. (2019). Efficient hyperparameter tuning with grid search for text categorization using KNN approach with BM25 similarity. Open Comput. Sci..

[15] Panigrahy S.K., Chiang K.N. Study on an artificial intelligence-based kernel ridge regression algorithm for wafer-level package reliability prediction. Proceedings of the IEEE 71st Electronic Components and Technology Conference (ECTC).

[16] Yin C., Zhu Y., Fei J., He X. (2017). A deep learning approach for intrusion detection using recurrent neural networks. IEEE Access.

[17] Chen J., Jing H., Yuan C., Liu Q. (2019). Gated recurrent unit-based recurrent neural network for remaining useful life prediction of nonlinear deterioration process. Reliab. Eng. Syst. Saf..

[18] Tang J., Deng C., Huang G.-B. (2016). Extreme learning machine for multilayer perceptron. IEEE Trans. Neural Netw. Learn. Syst..

[19] Tsiouris K.M., Pezoulas V.C., Zervakis M., Konitsiotis S., Koutsouris D.D., Fotiadis D.I. (2018). A long short-term memory deep learning network for the prediction of epileptic seizures using EEG signals. Comput. Biol. Med..

[20] Ghosh S., Ekbal A., Bhattacharyya P. (2023). Natural language processing and sentiment analysis: Perspectives from computational intelligence. Computational Intelligence Applications for Text and Sentiment Data Analysis.

[21] Law R.C., Cheang R., Tan Y.W., Azid I.-A. Thermal performance prediction of QFN packages using artificial neural network (ANN). Proceedings of the Thirty-First IEEE/CPMT International Electronics Manufacturing Technology Symposium.

[22] Subbarayan G., Li Y., Mahajan R.L. (1996). Reliability simulations for solder joints using stochastic finite element and artificial neural network models. J. Electron. Packag..

[23] Panigrahy S.K., Tseng Y.C., Lai B.R., Chiang K.N. (2021). An overview of AI-assisted design-on-simulation technology for reliability life prediction of advanced packaging. Materials.

[24] Kuo H.C., Chang C.Y., Yuan C.A., Chiang K.N. (2023). Wafer-level packaging solder joint reliability lifecycle prediction using SVR-based machine learning algorithm. J. Mech..

[25] Cheng H.C., Ma C.L., Liu Y.L. (2023). Development of ANN-based warpage prediction model for FCCSP via subdomain sampling and Taguchi hyperparameter optimization. Micromachines.

[26] Cheng H.C., Tai L.C., Liu Y.C. (2021). Theoretical and experimental investigation of warpage evolution of flip chip package during fabrication. Materials.

[27] Czyzewski J., Rybak A., Gaska K., Sekula R., Kapusta C. (2020). Modelling of effective thermal conductivity of composites filled with core-shell fillers. Materials.

[28] Cheng H.C., Li R.S., Lin S.C., Chen W.H., Chiang K.N. (2016). Macroscopic mechanical constitutive characterization of through-silicon- via-based 3-D integration. IEEE Trans. Compon. Packag. Manuf. Technol..

